# Roundabout receptor signaling: beyond axon guidance

**DOI:** 10.1038/s41392-026-02713-5

**Published:** 2026-05-12

**Authors:** Naresh L. Selokar, Wilfried A. Kues

**Affiliations:** 1 Friedrich-Loeffler-Institut, Federal Research Institute for Animal Health, Biotechnology/Stem Cell Unit, Neustadt Rbge, Germany; 2 Animal Biotechnology Division, ICAR-National Dairy Research Institute (NDRI), Karnal, India

**Keywords:** Reprogramming, Epigenetic memory

In a recent study published in Cell, Griffin et al.^1^ demonstrated that skin fibroblasts from distinct anatomic origins have different potentials for wound healing in mice, resulting in reduced scar formation in neural crest-derived skin compared to mesoderm-derived skin. Single-cell RNA sequencing revealed higher expression of *Robo2* in neural crest-derived facial fibroblasts, which suppressed pro-fibrotic gene transcription; accordingly, forced upregulation of the SLIT2-ROBO2 (Slit glycoprotein 2-roundabout receptor 2) pathway improved wound healing processes.

SLIT glycoproteins act as agonists for transmembrane roundabout (ROBO) receptors and were initially identified for being important for the correct guidance of axons and the development of the neuronal system. Recently, it has become clear that SLIT-ROBO signaling is also critical for several other processes, such as organ development, reproduction, and in cancer progression. In vertebrates, a family of SLIT glycoproteins, SLIT1, SLIT2, and SLIT3, has been identified so far, which is highly conserved across animal species. The multidomain proteins consist of a N-terminal region of four leucine-rich repeat domains, followed by seven to nine epidermal growth factor-like domains, a laminin G-like domain, and finally a C-terminal region of a cysteine-rich domain. The active N-terminal domain binds to transmembrane ROBO receptors (ROBO1, ROBO2, ROBO3, and ROBO4) and induces intracellular signaling, which is best studied in axon guidance.

**Fig. 1:**
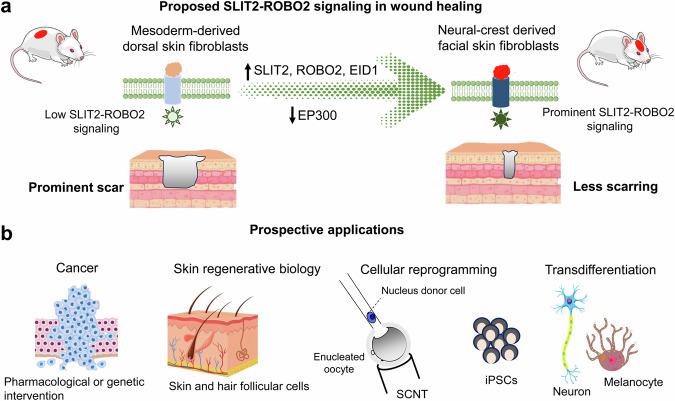
SLIT2-ROBO2 signaling and prospective applications. **a** Proposed SLIT2-ROBO2 axis in wound healing. **b** Prospective applications of genetic or pharmaceutical interventions in the SLIT-ROBO signaling. SCNT stands for somatic nuclear transfer, and iPSCs stands for induced pluripotent stem cells

Here, the authors assessed the wound healing in different anatomical locations of mice. The regions of the face that originate from the neural crest showed significantly reduced scarring after precise small skin excisions compared to regions of the scalp, back, and abdomen. Although not tested here, it has been suggested that the oral mucosa is more resilient to scar formation than craniofacial skin. Here, single-cell RNA sequencing showed particularly higher *Robo2* expression levels in the facial fibroblasts. The critical role of ROBO2 expression was substantiated by flow cytometric sorting of facial fibroblasts from transgenic mice with ubiquitous expression of the enhanced green fluorescent protein (EGFP) into ROBO2-positive and ROBO2-negative cells. The transplantation of ROBO2-positive fibroblasts into dorsal wounds indeed resulted in reduced scar formation, whereas the ROBO2-negative cells did not. The genetic EGFP reporter verified the successful transplantation of the facial fibroblasts. Together with additional experimental manipulations of molecules of the SLIT2-ROBO2 axis in vitro and in vivo (CRISPR-mediated knock-out, or overexpression), it turned out that this pathway signaled via EID (EP300-interacting inhibitors of differentiation)-mediated inhibition of EP300 to maintain a less differentiated cell state and a less fibrotic state (Fig. 1a). The EP300 is a broadly expressed histone acetyltransferase and transcriptional coactivator, involved in cell growth regulation and differentiation. Interestingly, this signaling axis could be pharmacologically targeted by the application of the small molecule I-CBP112, a bromodomain inhibitor. Bromodomain inhibitors are currently tested as potential candidates for anticancer therapies.^2^ I-CBP112 selectively inhibits the cAMP responsive-element binding protein (CREBBP) and the EP3000. The inhibitor has been shown to inhibit trimethylation of lysine 27 on histone H3 in human acute myeloid leukemia (AML) cells. Here, I-CBP112 application to dorsal skin injuries significantly improved wound healing and reduced scar formation. Whether the targeting of a central epigenetic player is suitable for systemic treatments warrants further research.

The results of this study open new avenues to explore the potential of neural crest skin-derived fibroblasts in reprogramming studies and regenerative applications. Fibroblasts are among the most commonly used cell types in reprogramming research, including somatic cell nuclear transfer (SCNT), induced pluripotent stem cell (iPSC) generation, transdifferentiation, and organoid development. Looking ahead, it is worth investigating the suitability of facial fibroblasts for SCNT outcomes, which currently achieve relatively low total efficiency (max. up to 5% of the reconstructed zygotes develop into healthy offspring).^3^ Notably, several studies have reported that fetal fibroblasts perform better than adult fibroblasts in SCNT outcomes and iPSC generation, paralleling findings that fetal skin exhibits superior scarless regeneration compared with adult skin.^4^ Furthermore, the use of EP300 inhibitor I-CBP112 has been reported to mitigate scar formation in dorsal wounds similar to facial skin. These observations suggest a compelling opportunity to prime fibroblasts with EP300 inhibitor(s) prior to SCNT experimentation or iPSC induction (Fig. 1b), potentially enhancing their overall reprogramming efficiency and regenerative potential.

In regenerative medicine, the clinical application of stem cells remains limited by complex culture conditions, high costs, tumorigenic properties, and ethical considerations. However, with the recent developments, including genome editing, it has become feasible to directly transdifferentiate skin-derived fibroblasts into specialized cell types, such as cardiomyocytes, neurons, hepatocytes, and melanocytes, without passing through a pluripotent state. The better ability of facial fibroblasts in wound healing processes further supports the exploration of these fibroblasts for regenerative applications. In addition to facial fibroblasts, embryonic neural crest lineages give rise to two major cell types, namely melanocytes and neurons. Previous work has shown that combinations of transcription factors, namely MITF, SOX10, and PAX3 were sufficient to reprogram fibroblasts into functional melanocytes.^5^ This study^1^ reported the high expression of SOX10 in facial fibroblasts compared with fibroblasts from other anatomical sites, suggesting an intrinsic neural crest–like signature in these cells. This encourages that facial fibroblasts might be more suitable to melanocytic transdifferentiation, potentially omitting the requirement for exogenous SOX10 or inclusion of EP300 inhibitors. A similar guiding approach could be applied to the direct conversion of fibroblasts into functional neurons in which SLIT-ROBO signaling plays significant role in neuron maturation and migration. Such directed reprogramming from facial fibroblasts could open multiple research avenues in the fields of clinical translations and neurobiology, including patient specific neuronal assays and cell-based therapies.

This study builds upon the understanding of SLIT-ROBO signaling and its role in wound healing following excisional injury in a murine model. Of course, the limitations of the murine model in relation to the anatomy of human skin must be considered. Human skin is thicker and more complex, with a robust epidermis of five to ten layers and a prominent dermal-epidermal boundary. Pig skin may be a better proxy for human tissue due to its striking similarities in anatomy, physiology, and biochemistry. Additionally, transplantation of human skin onto immune-deficient murine or porcine models could be performed to directly assess the role of SLIT-ROBO signaling in this tissue. Therefore, it is important to extend the study of this signaling pathway to animal models that more closely mirror the structure of human skin, as well as to other abnormal fibrotic conditions, such as keloids and scleroderma. In summary, SLIT-ROBO signaling plays a significant role in wound healing processes of mice; however, translation into therapies for humans will require an in-depth evaluation in the respective physiological complexities. Additionally, highly specific inhibitor molecules for the SLIT-ROBO axis must be developed to ensure clinical safety and efficacy.
